# MSC surface markers (CD44, CD73, and CD90) can identify human MSC-derived extracellular vesicles by conventional flow cytometry

**DOI:** 10.1186/s12964-015-0124-8

**Published:** 2016-01-12

**Authors:** Teresa L. Ramos, Luis Ignacio Sánchez-Abarca, Sandra Muntión, Silvia Preciado, Noemí Puig, Guillermo López-Ruano, Ángel Hernández-Hernández, Alba Redondo, Rebeca Ortega, Concepción Rodríguez, Fermín Sánchez-Guijo, Consuelo del Cañizo

**Affiliations:** Servicio de Hematología, IBSAL-Hospital Universitario de Salamanca, Paseo de San Vicente 58-182, 37007 Salamanca, Spain; Centro de Investigación del Cáncer, Universidad de Salamanca, Salamanca, Spain; Centro en Red de Medicina Regenerativa y Terapia Celular de Castilla y León, León, Spain; Instituto de Investigación Biomédica de Salamanca (IBSAL), Salamanca, Spain; Departamento de Bioquímica y Biología Molecular, Universidad de Salamanca, Salamanca, Spain

**Keywords:** Mesenchymal stromal cells, Flow cytometry, Extracellular vesicles characterization

## Abstract

**Background:**

Human mesenchymal stromal cells (hMSC) are multipotent cells with both regenerative and immunomodulatory activities making them an attractive tool for cellular therapy. In the last few years it has been shown that the beneficial effects of hMSC may be due to paracrine effects and, at least in part, mediated by extracellular vesicles (EV). EV have emerged as important mediators of cell-to-cell communication. Flow cytometry (FCM) is a routine technology used in most clinical laboratories and could be used as a methodology for hMSC-EV characterization. Although several reports have characterized EV by FCM, a specific panel and protocol for hMSC-derived EV is lacking. The main objective of our study was the characterization of hMSC-EV using a standard flow cytometer.

**Methods:**

Human MSC from bone marrow of healthy donors, mesenchymal cell lines (HS-5 and hTERT) and a leukemic cell line (K562 cells) were used to obtain EV for FCM characterization. EV released from the different cell lines were isolated by ultracentrifugation and were characterized, using a multi-parametric analysis, in a conventional flow cytometer. EV characterization by transmission electron microscopy (TEM), western blot (WB) and Nano-particle tracking analysis (NTA) was also performed.

**Results:**

EV membranes are constituted by the combination of specific cell surface molecules depending on their cell of origin, together with specific proteins like tetraspanins (e.g. CD63). We have characterized by FCM the EV released from BM-hMSC, that were defined as particles less than 0.9 μm, positive for the hMSC markers (CD90, CD44 and CD73) and negative for CD34 and CD45 (hematopoietic markers). In addition, hMSC-derived EV were also positive for CD63 and CD81, the two characteristic markers of EV. To validate our characterization strategy, EV from mesenchymal cell lines (hTERT/HS-5) were also studied, using the leukemia cell line (K562) as a negative control. EV released from mesenchymal cell lines displayed the same immunophenotypic profile as the EV from primary BM-hMSC, while the EV derived from K562 cells did not show hMSC markers. We further validated the panel using EV from hMSC transduced with GFP.

Finally, EV derived from the different sources (hMSC, hTERT/HS-5 and K562) were also characterized by WB, TEM and NTA, demonstrating the expression by WB of the exosomal markers CD63 and CD81, as well as CD73 in those from MSC origin. EV morphology and size/concentration was confirmed by TEM and NTA, respectively.

**Conclusion:**

We described a strategy that allows the identification and characterization by flow cytometry of hMSC-derived EV that can be routinely used in most laboratories with a standard flow cytometry facility.

**Electronic supplementary material:**

The online version of this article (doi:10.1186/s12964-015-0124-8) contains supplementary material, which is available to authorized users.

## Background

Human mesenchymal stromal cells (hMSC) are multipotent adult stem cells that are not only able of differentiate into most mesodermic cells [1] but also display a potent immunomodulatory activity [2, 3]. These two properties make them an attractive potential therapeutic tool for cell therapy programs. hMSC can be isolated from most tissues, although bone marrow (BM) and adipose tissue are the cell sources most extensively used [4]. Although hMSC have been successfully employed in many clinical trials to date, the ultimate underlying therapeutic mechanisms are still a matter of debate [5]. Pre-clinical studies demonstrated a minimal contribution in direct tissue repair (with the exception of bone regeneration) [6, 7]. Therefore, the beneficial effects of hMSC have been mostly attributed to the paracrine secretion of soluble factors [8]. In addition, it has been recently described that some of the effects of hMSC can be mediated through the release of extracellular vesicles [9, 10]. The exchange of EV is a complex and conserved mechanism of cell-to-cell communication. EV are a heterogeneous population that can be classified as exosomes or as shedding vesicles depending on their origin [8, 11, 12]. When the vesicle is formed from the inward of the endosomal membrane, it is designated as an exosome which has a small size (30–120 nm) and is secreted through exocytosis*.* Shedding vesicles are generated by budding from the plasma membrane. They are heterogeneous since their size (80–1000 nm) depends on their cell of origin as well as the releasing stimuli [13]. When released, EV can be incorporated into the recipient cell by direct contact with the cell membrane by their surface receptors or can be incorporated by membrane fusion [14]. EV can transfer important biological information to the recipient cells, such as surface receptors, proteins, mRNA, microRNA and bioactive lipids [15]. Structurally, EV membranes are enriched in cholesterol, sphingomyelin, and ceramide (lipid rafts) [16–19].

EV sub-populations express specific proteins like tetraspanins (CD81, CD63, and CD9) and others depending on their cell of origin [13, 20–22]. However it is difficult to distinguish different sub-types of EV due to their overlapping composition, density and size [10, 21], because of that we adopted the term EV suggested by the International Society for Extracellular Vesicles (ISEV) [23].

Bone marrow MSC-derived EV should express specific markers of mesenchymal lineage (e.g. CD44) and also of EV (e.g. CD63). Some of these molecules are critical for EV biological action (e.g. CD44) and should be present in these EV. The blockade of this latter cell surface glycoprotein deregulates vesicle uptake by tubular cells [24].

Flow cytometry (FCM) is a powerful methodology for EV detection and characterization [25] in most clinical laboratories. FCM is a technology that guides single particles through a laser beam in a hydro-dynamically focused fluid stream. The properties measured include particle’s relative size, granularity or internal complexity, and relative fluorescence intensity. These characteristics are determined using an optical-to-electronic coupling system that records how the cell or particle scatters incident laser light and emits fluorescence.

In general, the methodology is divided into two steps. The first one is based on their side and forward scattering intensity, where the selection of the threshold value is of extraordinary relevance. The detection of EV by FCM used in most laboratories is a challenge since conventional flow cytometers are not prepared to detect events below 500 nm [26]. The second step for EV detection is based on their specific labeling with fluorescent ligands.

The emergence of hMSC-derived EV (hMSC-EV) as a new and potentially effective strategy in regenerative medicine [27] has raised the need of more specific detection and characterization protocols. Although several reports have characterized EV by FCM, a specific panel and protocol for hMSC-EV FCM analysis is lacking. The main objective of our study was to optimize hMSC-EV-characterization methodology by FCM.

## Methods

### hMSC isolation, expansion and characterization

Bone marrow (BM) human mesenchymal stromal cells (hMSC) were isolated from 5 healthy donors (three males/two females) with a median age of 48 years (range 21–49 years). In all cases, written informed consent was previously obtained according to institutional guidelines and approved by the Comite Etico de Investigacion Clinica del Area de Salud de Salamanca (located in Salamanca, Spain) with reference number 70/07/2015. Mononuclear cells (MNC) were isolated from fresh BM aspirates and separated by density gradient centrifugation (Ficoll-Paque, GE Healthcare Bio-Sciences, AB, Uppsala, Sweden) and cultured in standard culture medium, as previously described [7]. Cells were cultured at 37 °C in a humidified atmosphere with 5 % of carbon dioxide. At passage 3, hMSC characterization was performed according to the recommendations of the International Society for Cellular Therapy (ISCT) [28]. Isolation of hMSC-EV were performed until passage number 6.

### hMSC transduction

For transduction of hMSC we used a third generation of replication-defective self-inactivating lentiviral vector. A 1.7 kb luciferase (Luc2) fragment was isolated from pGL4.10 (*Luc2*) vector (Promega, Madison, WI) using XhoI and NheI restriction sites. Subsequently, the *Luc2* fragment was cloned in the LV pRRL-cPPT-CMV-IRES-GFP-PRE-SIN (kindly provided by Prof. Hoeben, Leiden University Medical Center, Leiden, Netherlands [46]) using the same restriction sites, downstream of the CMV promoter, to generate pRRL-cPPT-CMV-Luc2-IRES-GFP-PRE-SIN. The protocol of hMSC transduction performed following procedures as previously reported [47] [48]. In this study we used hMSC-GFP^+^ transduced with efficiency over 60 %.

### Cell lines

We isolated EV from human stromal cell lines to compare them to EV derived from primary hMSC. The stromal cell lines used were: hMSC-TERT, a hMSC cell line, immortalized by expression of the telomerase reverse transcriptase gene described by Mihara K et al. in 2003 [49], that was a generous gift from Dr. Dario Campana (Department of Oncology and Pathology, St Jude Children’s Research Hospital, Memphis, TN, USA) and the fibroblastoid cell line HS-5, immortalized from human bone marrow using the E6 and E7 human papillomavirus genes, purchased from ATCC (ATCC® CRL11882™).

We also used K562 cells (a human cell line derived from a patient with Philadelphia translocation-Ph^+^- blast crisis) purchased from ATCC (ATCC® CCL243™), as a negative control for immunophenotypic assays.

These cell lines were grown in Dulbecco’s modified Eagle’s medium (DMEM; Gibco, Paisley, UK) (hMSC-TERT and HS-5) or RPMI 1640 medium (DMEM; Gibco, Paisley, UK) (K562) supplemented with 10 % fetal bovine serum (FBS, Gibco, Paisley, UK), 100 U/mL penicillin and 100 mg/mL streptomycin (Gibco, Paisley, UK). All cell types were cultured at 37 °C in a humidified atmosphere in the presence of 5 % CO_2_–95 % air.

### Preparation of conditioned medium and isolation of EV

EV were purified from the supernatants of hMSC (from healthy donors), hMSC–GFP^+^, hMSC-TERT, HS-5 and K562 cell lines. Briefly, cells were washed with phosphate–buffered saline (PBS) and then were cultured in RPMI with antibiotic and deprived of FBS. After incubation for 24 h, the conditioned medium was collected and centrifuged at 2000 *g* for 20 min at 4 °C to remove the cell debris. In some cases, mainly when the supernatant was obtained from many culture flasks, this supernatant was filtered through a 0.22 μm filter (Millipore, Billerica, MA) to remove debris. Then, the conditioned medium (CM) was ultracentrifuged at 100,000 *g* for 70 min at 4 °C. The pellets were then washed with doubled filtered PBS and ultracentrifuged at 100,000 *g* for 70 min at 4 °C and suspended in PBS (doubled filtered). All the samples were ultracentrifuged in polycarbonate tubes (25 mm × 89 mm, Beckman Coulter) that have a volume of 22 ml. A Beckman Coulter ultracentrifuge (Beckman Coulter OptimaL-90K ultracentrifuge; Beckman Coulter, Fullerton, CA, USA) was used with a fixed angle rotor type 70ti.

### Multiparametric flow cytometry analysis

The following monoclonal antibodies (mouse anti-human) were used for flow cytometric immunophenotyping of hMSC-EV and hMSC: CD90 FITC (Fluorescein isothiocyanate), CD34 FITC, CD44 PE (phycoerythrin), CD73 PE, CD14 PE, CD63 PE, CD166 PerCP-Cy 5.5 (phycoerythrin-cyanine 5.5), CD34 PerCP-Cy5.5, CD45 PerCP-Cy5.5, HLA-DR PerCP-Cy5.5, CD19 PerCP-Cy5.5, CD81 APCH7 (APC-cyanine tandem dye), CD45 V500 (BD Horizon V500), CD44 APC (Allophycocyanin) and CD105 APC. All of them were purchased from BD Biosciences (San José, CA) except for CD44 APC, Cytognos (Salamanca) and CD105 (R&D Systems, Minneapolis, MN, USA) purchased from Cytognos and R&D systems, respectively. We used a FACSCalibur flow cytometer (BD Biosciences) for hMSC sample acquisition. The FACSCalibur flow cytometer is a 4-color instrument with two lasers, blue (488-nm) and red (633 nm). Labeled cells were acquired immediately after the staining using a FACSCalibur flow cytometer equipped with the CellQuestTM program (BD Biosciences).

Human MSC were characterized using the following conjugated monoclonal antibody combinations: CD34 FITC/CD73 PE/CD45 PerCPCy5.5/CD105, CD44 FITC/CD14 PE/CD19 PerCPCy5.5 and CD90 FITC/CD166 PE/HLA-DR PerCPCy5.5. Human MSC (5 × 10^5^ cells) were suspended in PBS and incubated with combinations of the monoclonal antibodies described above and also was acquired hMSC unstained used as control.

We used a FACSCanto II flow cytometer (BD Biosciences San Jose, CA) for EV acquisition using the FACSDiva 6.1 software (BD Biosciences). The FACSCanto II flow cytometer is an 8-color instrument with 3 lasers, blue (488-nm, air cooled, 20-mW solid state), red (633 nm, 17-mW HeNe) and violet (405 nm, 30-mW solid state).

For FACSCanto II flow cytometer calibration, we used SPHERO™ Rainbow Calibration Particles (Rainbow Calibration, eight peaks-Spherotech, Inc. Lake Forest, USA), following the recommendation of the EuroFlow consortium [50], with adaptation of light scatter detectors channels to properly identify the EV. For fluorescence compensation, BD™ CompBeads (BD Biosciences, San José, CA, USA) were used with generic and with the fluorochrome-label antibodies used in our experiments. Samples were acquired after daily evaluation of instrument’s performance using the Rainbow beads particles. These particles were also used to identify the *electronic noise* or background, which comes primarily from extraneous particles reaching the detector (light scatter) [9]. The samples were acquired after the cytometer was calibrated and compensated.

**Fig. 1:**
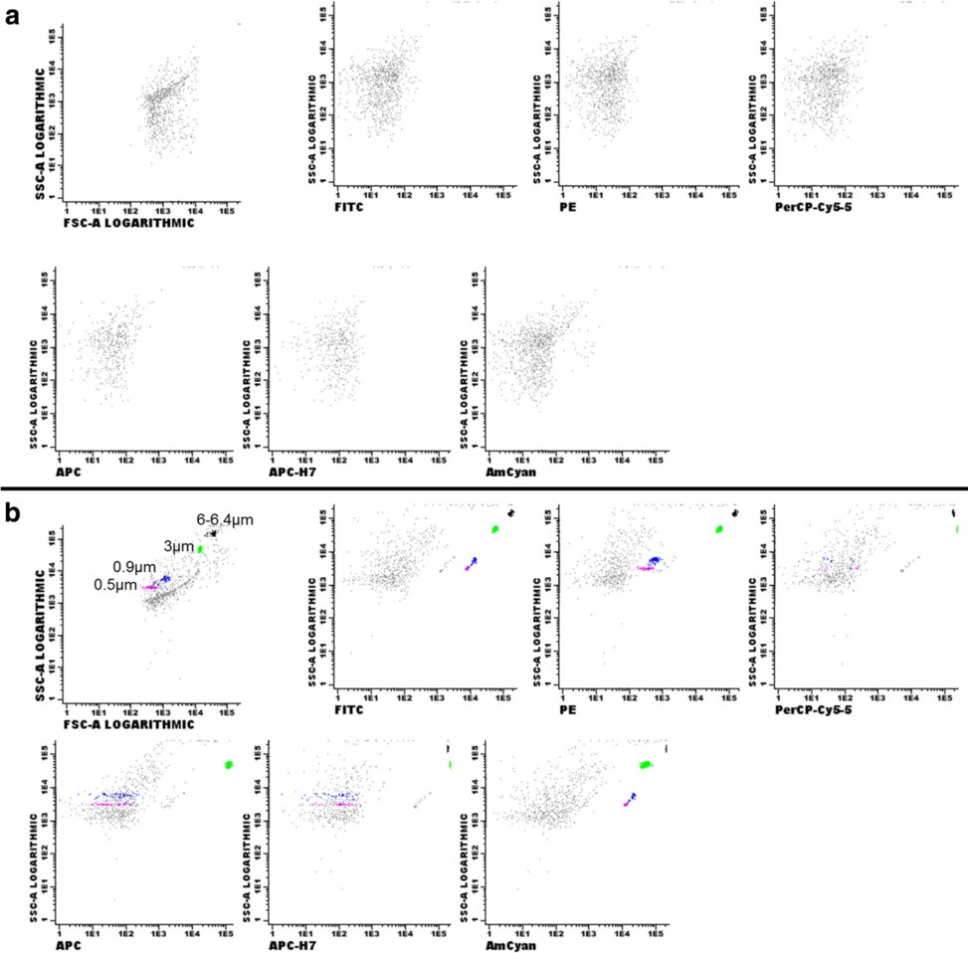
Representative FCM dotplot showing acquired PBS 0.22 μm doubled filtered (**a**) and PBS doubled filtered with beads (0.5, 0.9, 3, 6–6.4 μm) (**b**). All samples used in this work were acquired when positive events were not present in the different fluorescence channels in the acquired PBS (*double filtered*)

Before EV acquisition, the instrument was washed with rinse solution and with double filtered PBS (through 0.2 μm membrane Millipore filters) after 6 h of sedimentation, to reduce the instrument background noise (Fig. 1). The forward scatter component (FSC) parameter is used to determine the EV size. Double-filtered PBS was acquired with a mix of fluorescent beads composed of varied diameters (0.5, 0.9 and 3 μm) Megamix (Biocytex, Marseille, France) and perfect-Count Microespheres (Cytognos, Salamanca, Spain) of 6–6,4 μm in size. The beads of different sizes were used in all of the experiments, with this approach we validated our instrument basis on the capacity to discriminate between 0.5 and 0.9 μm Megamix beads using the FSC parameter, as well as their background noise. The acquisition of the samples was only performed when the number of double-filtered PBS events acquired per second ranged between 25 and 50 at low speed with threshold settings between 300 and 350. EV recovered from ultracentrifugation were suspended in double filtered PBS and stained by direct immunofluorescence using monoclonal antibodies. For the study of antigen expression, samples were incubated in the dark with the appropriate combination of monoclonal antibodies during 15 min. For the optimization of the immunophenotypic characterization of EV released from hMSC by FCM, in the immunophenotypic panel hMSC markers (CD90, CD44 and CD73), negative markers of hMSC (CD34 and CD45) and EV markers (CD81 and CD63) were included. Samples were incubated for 15 min in the dark using an 6-color combination, set up with the following monoclonal antibody panel: 5 μl of anti-CD90-FITC/ 10 μl of anti-CD73-PE or anti-CD63-PE/10 μl of anti-CD34-PerCPCy5.5/5 μl of anti-CD44-APC/ 5 μl of anti-CD81-APCH7/5 μl of anti-CD45-V500. After incubation the excess of antibodies was washed with double filtered PBS at 2000 g for 10 min. The final volume (suspended cells, MoAbs, PBS and 500 μl Megamix beads) was 700 μl.

To analyze if the antibodies indicated above do not form aggregates we used as negative control the double-filtered PBS containing each single antibody as well as the combination of all the antibodies used. The doubled filtered PBS was stained following the same methodology as EV (Additional file 1: Figure S2 and Additional file 2: Figure S3). Data were acquired immediately after the staining. In all samples, EV not labeled with the different antibodies were used to discriminate the positive and the negative population.

A total of 100,000 events were acquired (at low speed). Data were analyzed using the Infinicyt program (Cytognos, Salamanca, Spain).

### Immunoblotting

Ultracentrifuged EV were re-suspended in lysis buffer (25 mM HEPES, pH 7.5, 150 mM NaCl, 1%Igepal, 10 % glycerol, 10 mM MgCl2, 1 mM EDTA, 25 mM NaF, 1 mM Na2VO4, plus proteinase inhibitors), and incubated 20 min on ice. Non-soluble material was eliminated by centrifugation. Protein concentrations were determined using the Bradford assay. Samples were then subjected to SDS-PAGE and the proteins were transferred onto Polyvinylidene fluoride (PVDF) membranes from GE Healthcare (Barcelona, Spain), as previously described [51]. Non-specific binding was blocked with 5 % non-fat dry milk. Primary antibodies were rabbit α-CD63 and α-CD81 (Exo AB Antibody Kit – System Biosciences) and rabbit α-CD73 (ab124725, Abcam Inc.). Incubation with the primary antibodies at the appropriate dilution was performed overnight at 4 °C. As secondary antibody an anti-rabbit IgG horseradish peroxidase-conjugated from Amersham Biosciences was used. Incubation with the secondary antibody was performed at room temperature for 1 h. Blots were visualized using chemiluminescence using ECL-Plus reagent (Amersham Biosciences).

### Transmission electron microscopy

EV pellets were re-suspended in PBS and fixed in 2 % paraformaldehyde (PF) and 2.5 % glutaraldehyde and stained with uranyl-oxalate solution. The images were captured using a transmission electron microscopy (FEITecnai G2 Spirit Biotwin) using a digital camera (Morada, Soft Imaging System, Olympus).

### Nano-particle tracking analysis

Analysis of absolute size distribution of EV was performed using NanoSight NS 300 (NanoSight Ltd., UK) equipped with a sCMOS camera. This technology is based on the assessment of both light scattering and Brownian motion to obtain the size distribution and concentration measurement of particles in liquid suspension. After isolation, the EV were diluted in 1 ml of doubled filtered PBS. The NTA measurement conditions were as follows: temperature between 21 and 23.6 °C; viscosity between 0.9 and 0.965 cP, frames per second 25, measurement time 60s. Before acquisition the samples were diluted in water (1:1000) due to their high concentration. The detection threshold was similar in all samples. The results indicate the mean sizes and standard deviations of at least three individual measurements.

## Results

### hMSC definition criteria

**Fig. 2:**
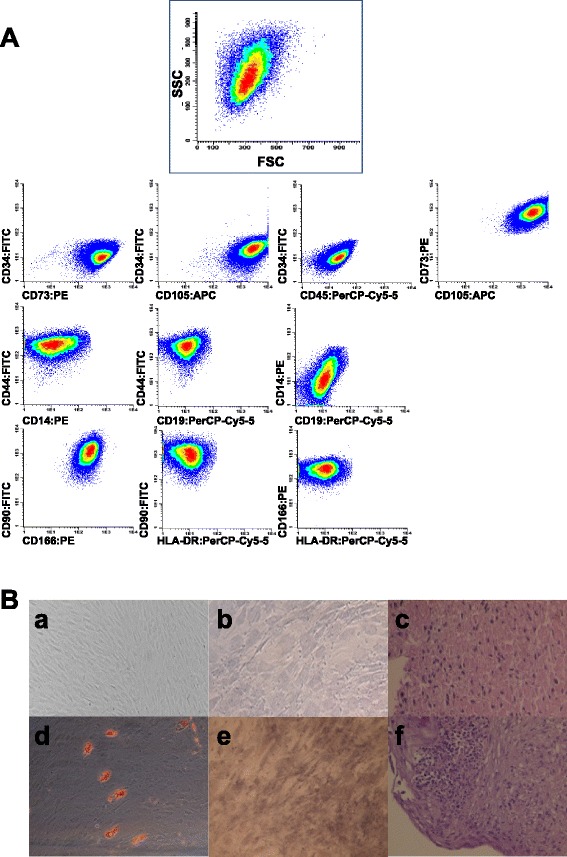
Characterization of human MSC. **a** Representative dot-plots of stained hMSC. **b** In vitro differentiation of hMSC to adipocytes (*a, d*), osteoblast (*b, e*) and chondrocytes (*c, f*) with differentiation medium (*d, e, f*) compared to control (*a, b, c*)

hMSC were isolated and expanded from BM samples and further characterized according to ISCT minimal definition criteria [28]. These cells did not express hematopoietic lineage markers such as CD14, CD19, CD34, CD45 and HLA-DR, and were positive for CD73, CD90, CD105, CD166 and CD44 thereby demonstrating a characteristic immunophenotype of hMSC In addition, their multilineage differentiation ability (into osteogenic, adipogenic and chondrogenic lineages) was demonstrated (Fig. 2). HTERT and H-S5 were also inmunophenotypically characterized using the same panel used for hMSC from BM.

### hMSC–EV flow cytometry analysis

EV were purified by ultracentrifugation collected from the three cell lines (HTERT, HS-5 and K562) and primary BM-hMSC, including hMSC-GFP^+^. After EV isolation, all the samples were labeled with the same panel of monoclonal antibodies. To identify EV obtained from hMSC, the mesenchymal markers CD90, CD44 and CD73 were used. The tetraspanin CD81 and CD63 were used as EV markers.

**Fig. 3:**
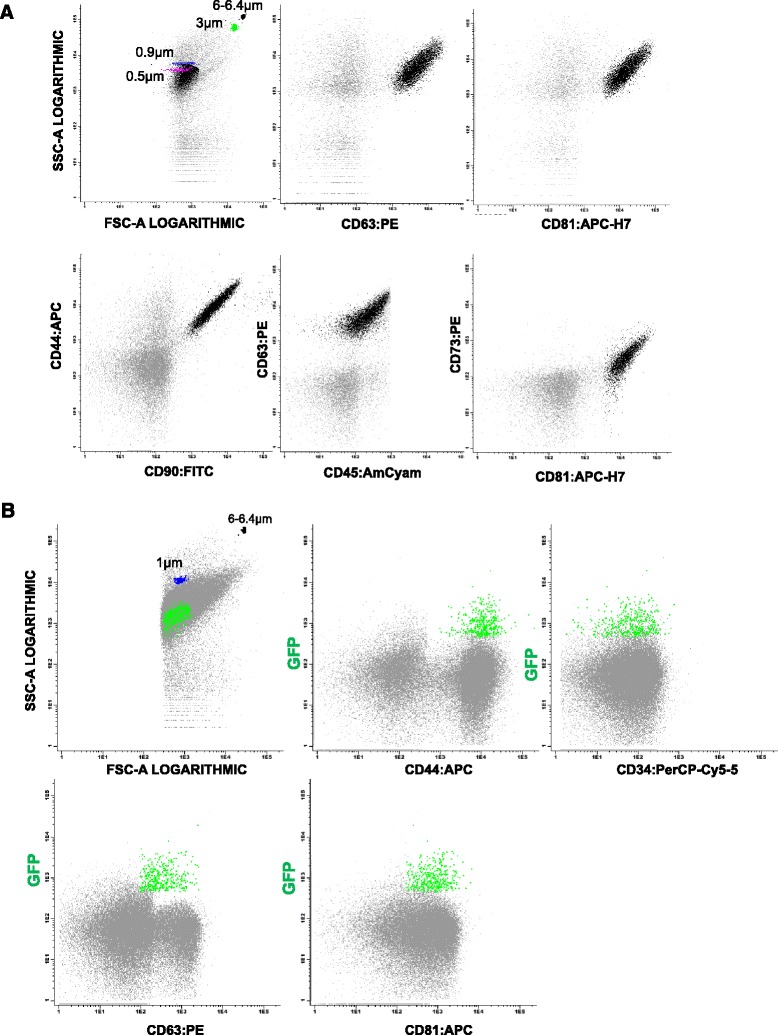
Multiparametric analysis of EV-hMSC. **a** Representative FCM dotplots showing the gate protocol for hMSC-EV. The gate of EV was defined by use of microbeads. Standard microbeads with a diameter of 0.9 μm were used to set the upper size limit for the EV and were used to gate the hMSC-EV. hMSC-EV stained with hematopoietic markers (CD34 and CD45) were negative and were positive for hMSC markers (CD90, CD44, CD73) and for EV markers (CD81 and CD63). In gray represents the control (unstained EV) and in black the EV stained with the different antibodies. **b** Representative dotplot of EV from hMSC-GFP^+^, in these case we first gate the particles that were positive in fluorescence 2 (GFP) and were also smaller than the upper size (0.9 μm). Then we confirmed with these gate that the EV from hMSC-GFP^+^ were positive for mesenchymal markers, negative for hematopoietic markers and positive for CD63 and CD81 (EV markers)

We were able to characterize EV released from BM-hMSC (Fig. 3), and in order to separate true events from background noise, EV were defined as particles that were less than 0.9 μm of diameter and were positive for the specific markers used. Dot plot images showed that EV were positive for CD90, CD44 and CD73, thus demonstrating that hMSC were the cell of origin (Fig. 3a). Because there are no specific markers for hMSC but a combination of positive and negative markers, we used CD34 and CD45 (hematopoietic markers) to demonstrate the specificity of monoclonal labeling. As shown in the dot plots, hMSC- EV were negative for these hematopoietic markers. Our results (Fig. 3a) showed that besides being positive for mesenchymal markers, negative for hematopoietic markers and with a size less than 0.9 μm, hMSC-EV were also positive for CD63 and CD81.

In our study we also used BM-hMSC transduced with a lentiviral vector containing the GFP gene. Once transduced, the cells express GFP that can be detected by FCM. Our results (Fig. 3b) showed that EV from hMSC-GFP^+^ also express GFP. Performing a live-gate on the positive events in the fluorescence-1 channel (where GFP is detected), we showed that this population was also positive for CD73 and CD44 (hMSC marker), and for CD81 and CD63 (EV marker). At the same time this population was negative for hematopoietic markers with sizes smaller than 0.9 μm.

**Fig. 4:**
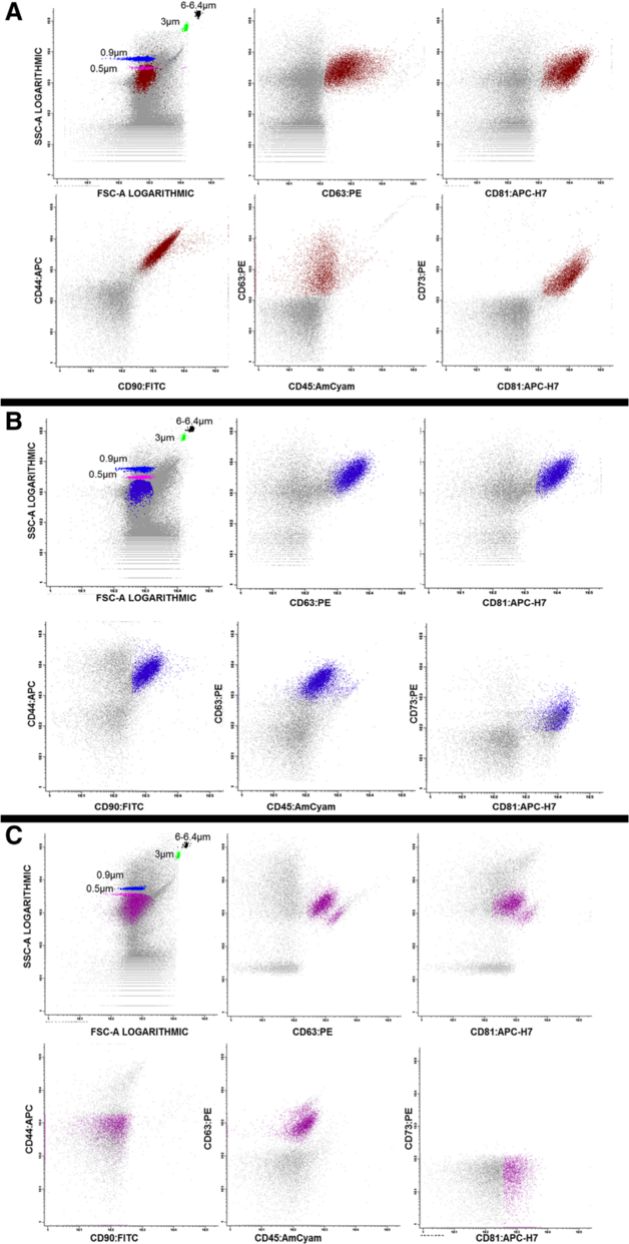
EV analysis of the different mesenchymal cell lines (hTERT and HS-5) and leukemia cell line (K562) the data is represented in dotplots. EV stained with hematopoietic markers (CD34 and CD45), positive markers for hMSC (CD90, CD44, CD73) and for EV markers (CD81 and CD63). **a** EV-hTERT cells. **b** EV- HS-5. **c** EV-K562

To confirm that this immunophenotypic panel was specific to characterize hMSC–EV, EV from three cell lines were analyzed: hTERT and HS-5 that are human mesenchymal cell lines and K562 that is a leukemia cell line used as negative control. As shown in Fig. 4, EV isolated from conditioned medium of hTERT and HS-5 cell lines, expressed the same immunophenotypical profile than EV from BM-hMSC. However EV obtained from K562 cell line did not show hMSC markers.

The use of controls in flow cytometry for EV characterization is very important. In a set of experiments to ensure that the population analyzed are EV and no other signals we acquired only Megamix (Additional file 3: Figure S1), doubled filtered PBS stained with each different antibody in an individual tube (Additional file 1: Figure S2), doubled filtered PBS with the combination of all the different antibodies (Additional file 2: Figure S3), unstained EV-hMSC and stained with each different antibodies in an individual tube (Additional file 4: Figure S4 and Additional file 5: Figure S5).

### Characterization of hMSC-EV

**Fig. 5:**
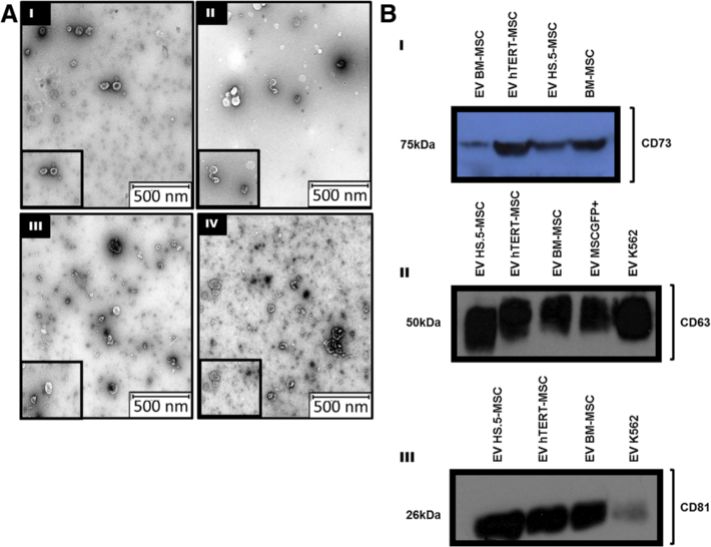
Representative images for validation of EV using TEM (**a**) and Western Blot (**b**). **a** I-EV-K562 cells. *II*-EV- BM-hMSC. *III*-EV-HS-5 and *IV*-EV-hTERT mesenchymal cell lines. Scale bar 500 nm. Original magnification: ×8000. **b** WB analysis of human mesenchymal protein CD75 (*I*) and human exosomal CD63 and CD81 proteins (*II* and *III*, respectively)

The morphology of EV was confirmed by phase-contrast transmission electron microscopy [29]. We observed typical bilayer-membrane vesicles with heterogeneous size from isolated EV of BM-hMSC and MSC cell lines (Fig. 5a). K562-EV also showed the characteristic rounded morphology with a hypodense center. Western blot analysis showed that EV from all conditioned media expressed the specific markers of EV (CD63 and CD81) and the EV from MSCs were also positive for the mesenchymal marker CD73 (Fig. 5b).

**Fig. 6:**
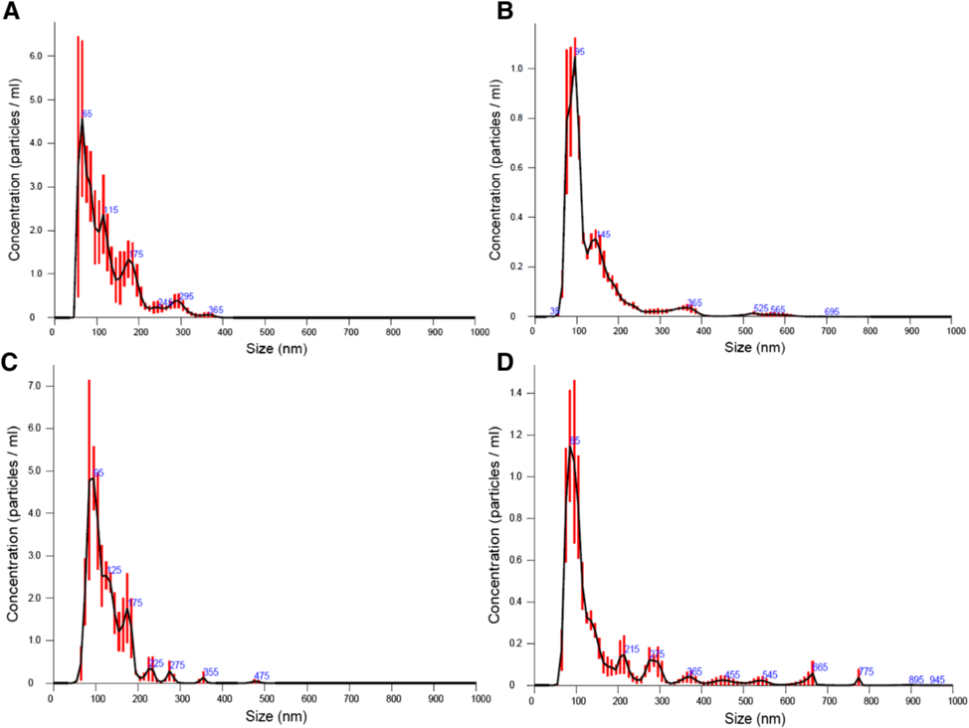
EV quantification using Nanosight nanoparticle tracking analysis. hMSC cells produced EV in cell culture with a mean size of 136 ± 86,8 nm, with a homogenous EV population. Cell lines hTERT and HS.5 (mesenchymal lines) and K562 cells (Leukemia cell line) also produce EV with size similar to EV–hMSC **a** - EV-K562; **b** - EV-BM-hMSC; **c** - EV-HS-5; **d** - EV-hTERT

Next, we characterized EV from different sources to determine their size with an independent method, nanoparticle tracking analysis. High particle concentration was detected in all samples (Table 1) being highest in hTERT and hMSC-EV. Size was homogenous between the different EV samples (Fig. 6). NTA estimated that the size of EV-hMSC was between 35 and 695 nm.

**Table 1:** Mean size and concentration of EV from HS.5, hTERT, K562 and hMSC culture measured by NTA

EV	Concentration (particles/mL)	Size (nm)
HS.5	3,29 × 10^11^ ± 2,4 × 10^10^	Mean – 125.6
		SD – 49.8
hTERT	7,64 × 10^11^ ± 4,62 × 10^10^	Mean – 159,7
		SD – 130,4
K562	3,36 × 10^11^ ± 5,05 × 10^10^	Mean – 122
		SD – 65,9
hMSC	6,40 × 10^11^ ± 1 × 10^10^	Mean – 136,6
		SD – 86,8

*SD* standard deviation calculated by NTA software. Numbers represent average values ± standard deviation (*n* = 3 measurement)

These results are in agreement with the current recommendations of the International Society for Extracellular Vesicles (MISEV) [30, 31].

## Discussion

Human MSC have emerged as one of the most promising cell type with potential clinical applications in cellular therapy programs [32]. Besides the direct differentiation potential into some cell types (e.g. osteoblasts for bone repair) [33], they exert their therapeutic effect mostly by paracrine secretion of cytokines and other mediators [8]. In addition, in the last few years it has been shown that hMSC also act through the secretion of EV. The use of MSC-derived EV has potential advantages over the use of the cells, including the theoretical absence of risk of malignant transformation and less regulatory hurdles for clinical application, since it is a cell-free therapeutic product [34].

EV can be isolated by different methods, and their characterization in most experiments is based on their structure/morphology by TEM, or on the tetraspanin expression by Western blot. There are other additional methods that have been applied to the detection and quantification of EV, including atomic force microscopy (AFM) [35], dynamic light scattering (DLS) [36], nanoparticle tracking analysis [37, 38] or resistive pulse sensing (RPS) [29]. Nevertheless, there is a need for a fast, reproducible and generalized method for identification of EV that takes into account not only their size but also their cell of origin.

For EV quantification the methodologies most widely used are NTA and FCM. The studies that compare these two methods discuss that they cannot be interchangeable since the size range that they can analyze is different. NTA allows obtaining and comparing size distribution mainly in particles less than 300 nm, what gives more accurate counts compared with FCM. However, FCM is a powerful technique for multiparametric analysis of single biological particles allowing the differentiation of distinct cellular origins [25, 29, 39].

In our opinion, FCM has the potential to characterize EV, but the identification of hMSC- EV is not a currently standardized process. We have used in the current work a flow cytometer FACSCanto II maintaining the standard cell based setup of the instrument unmodified as much as possible, to assess the potential viability of this type of measurements in a clinical routine used instrument. Thus, we established a protocol to provide an identification strategy that could be easily followed in most clinical institutions. The use of FCM for EV characterization has some limitations, because the method presented herein only allows analysis of EV above the threshold of detection of the instrument, and potentially excludes smaller particles below this threshold [40, 41]. In the last years, different groups have generated protocols of standardization in order to improve instrument performance and resolution. This new high sensitive flow cytometer with high light-scatter sensitivity, an optimized laser beam shaping and detectors are an improvement over the standard flow cytometers, which were unable to detect small particles. Several studies have reported other methodologies to detect EV by FCM, fluorescence-triggered detection is one of them. They demonstrated several advantages over light-scatter triggering signals with highly sensitive of EV detection.

There are a number of questions that need to be considered for an adequate identification and characterization of MSC-EV by FCM, which will be briefly discussed in the next paragraphs.

The first one is related to the selection of the monoclonal antibodies to be included in the identification panel. In this regard, we have selected antibodies that identify tetraspanins that are typically expressed by exosomes (CD81, CD63) [42], together with antibodies that recognized markers expressed by MSC in their membrane (CD90, CD44 and CD73) [43] and those that are consistently negative in MSC and positive in hematopoietic stem cells or endothelial cells (CD34) or in leukocytes (CD45). With this strategy we can differentiate by FCM those EV derived from MSC from those derived from HSC or from any leukocyte [44].

The second important factor for the identification of EV by FCM is the inclusion of two sets of microbeads of different sizes (a Megamix Kit of 0.5, 0.9 and 3 μm and a perfect-Count Microespheres of 6–6,4 μm in size). The inclusion of microbeads helps not only to define the upper forward scatter EV size gate limit, but this strategy also allows to correctly define the threshold level necessary to strictly acquire EV avoiding the instrument *background noise* [26, 45]*.*

The third important issue is to adequately confirm the calibration and the right compensation of the flow cytometer before initiating the acquisition of EV. The signal acquired by FCM of small particles is not only influenced by particle size but also by their refractive index, surface roughness, shape and possible light observation. Many efforts are aimed to EV standardization measurements by FCM for their correctly identification that include sample preparation, immunostaining and particle size-calibration protocols [41].

To minimize the *background noise* it is critical to acquire the data at low flow speed to increase the resolution as well as for the accurate discrimination between non-fluorescence noise and fluorescence signals. High flow speed increases the diameter of the sample stream that lets the particles move outside the center of the laser spot decreasing the resolution. Another critical issue to reduce the background noise is the use of 0.22 μm filtrated PBS, which reduces the number of small events recorded by FCM [29].

Controls are the most important and relevant information required to ensure that we detected EV and no unspecific signals. We performed a set of control assays to ensure the correct EV detection. Because we used FCM for EV detection with a closer methodology used in the mainly clinical research facilities some limitations were detected. One of them is that not all the EV population was detected and characterized because we only detected EV with size between 0.3 and 0.9 μm. NTA assays reveal the presence of a EV population with sizes inferior to 0.3 μm, which reveals that not all the EV population by our FCM are detected. Other limitation identified during control assays is the fact that EV are very small, we cannot be sure that the signal of one particle in one laser really corresponds at the same particle under the second laser that can be wrongly computed. One way of overcoming this problem is the use the fluorescence triggering approach.

To further confirm that our FCM identification strategy we have isolated EV from BM-hMSC transduced with a lentiviral vector containing the GFP gene, to unequivocally demonstrate that our protocol identifies these EV by the GFP expression. Although some authors use a cell tracker (e.g. Vibrant Cell, DiD or similar) for identification of EV and also for EV tracking in cellular incorporation studies, their use for FCM characterization may generate positive events due to the fluorescence overlap in other channels. Once transduced, the cells express GFP that can be detected by FCM avoiding the false positive events.

In addition to these confirmatory experiments, we have tested our strategy not only in primary hMSC-derived EV but also on EV secreted by two profusely employed mesenchymal stromal cell lines, hTERT and HS-5. EV derived from both cell lines can be easily identified by FCM following the protocol described in our study. By contrast, EV from one leukemia cell line showed a different expression profile.

## Conclusions

We have described a methodology to identify and characterize by flow cytometry hMSC-derived EV. The method is reproducible and reliable, and may be easily applied for EV characterization in most institutions and experimental settings.

## Additional files

Additional file 1: Figure S2. Representative FCM PBS dotplots. Doubled filtered PBS mixed with one antibody per tube. (TIF 528 kb) Additional file 2: Figure S3. Multiparametric FCM PBS dotplots. Doubled filtered PBS mixed with a combination of all different antibodies. (TIF 315 kb) Additional file 3: Figure S1. Detection by FCM of different size beads. Dotplot of Megamix (0.5, 0.9 and 3 μm) and 6–6.5 μm beads dotplot (A) and density histogram (B). (TIF 120 kb) Additional file 4: Figure S4. Unstained hMSC-EV dotplots. (TIF 494 kb) Additional file 5: Figure S5. Representative individual dotplots of hMSC-EV. (A) Dotplots of hMSC-EV stained with each different antibody per tube. (B) Mean fluorescence intensity (MIF) values of the different channels. (TIF 633 kb)

## Abbreviations

BM: bone marrow
EV: extracellular vesicles
FCM: flow cytometry
FSC: forward scatter component
GFP: green fluorescence protein
hMSC: human mesenchymal stromal cells
SSC: side scatter component
TEM: transmission electron microscopy
