# Supplementary figures and images for: MSC surface markers (CD44, CD73, and CD90) can identify human MSC-derived extracellular vesicles by conventional flow cytometry
Source: Cell Commun Signal. 2016 Jan 12;14:2. doi: 10.1186/s12964-015-0124-8 (PMC4709865; doi:10.1186/s12964-015-0124-8)

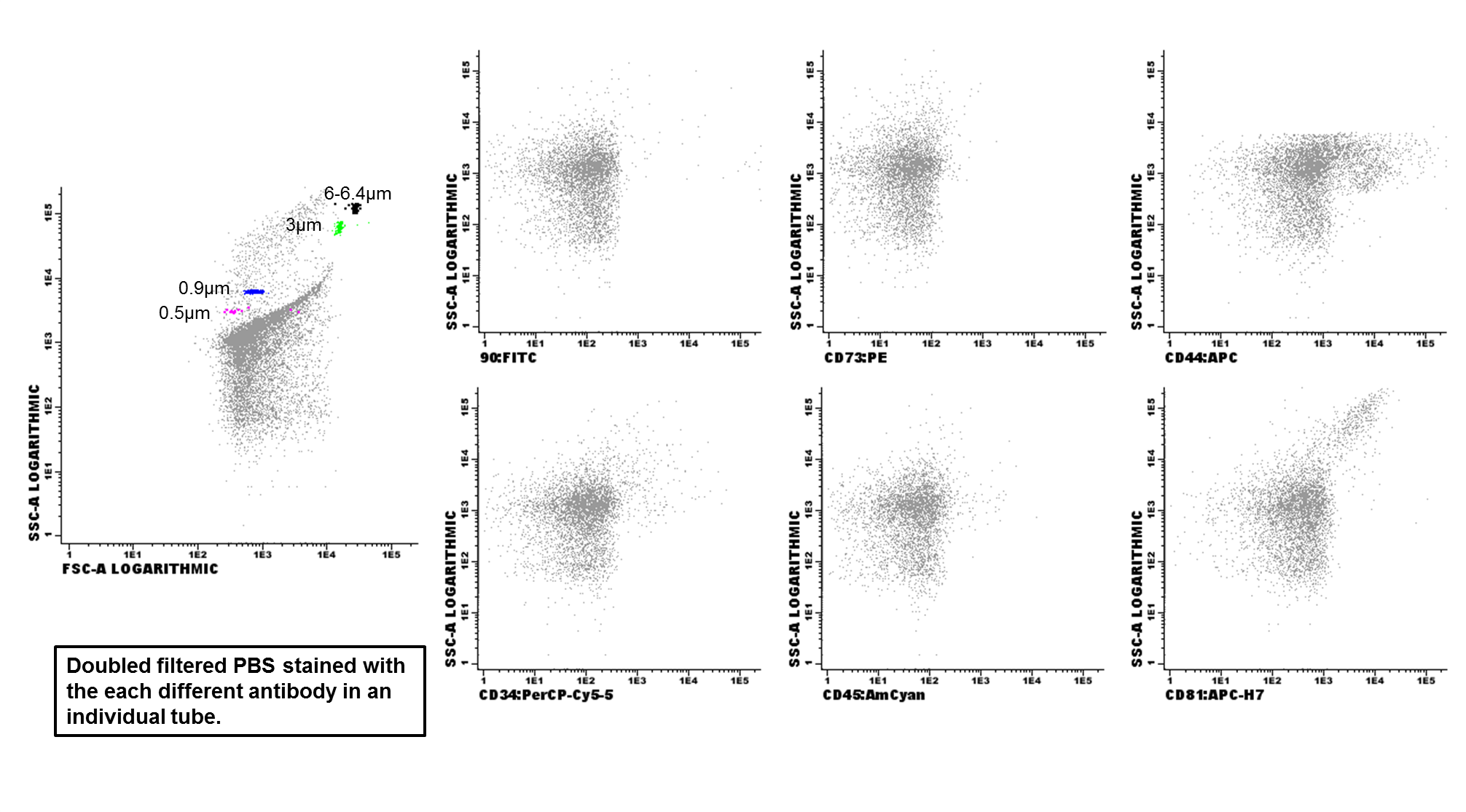

Supplement: Additional file 1: Figure S2. — Representative FCM PBS dotplots. Doubled filtered PBS mixed with one antibody per tube. (TIF 528 kb) [file 12964_2015_124_MOESM1_ESM.tif]

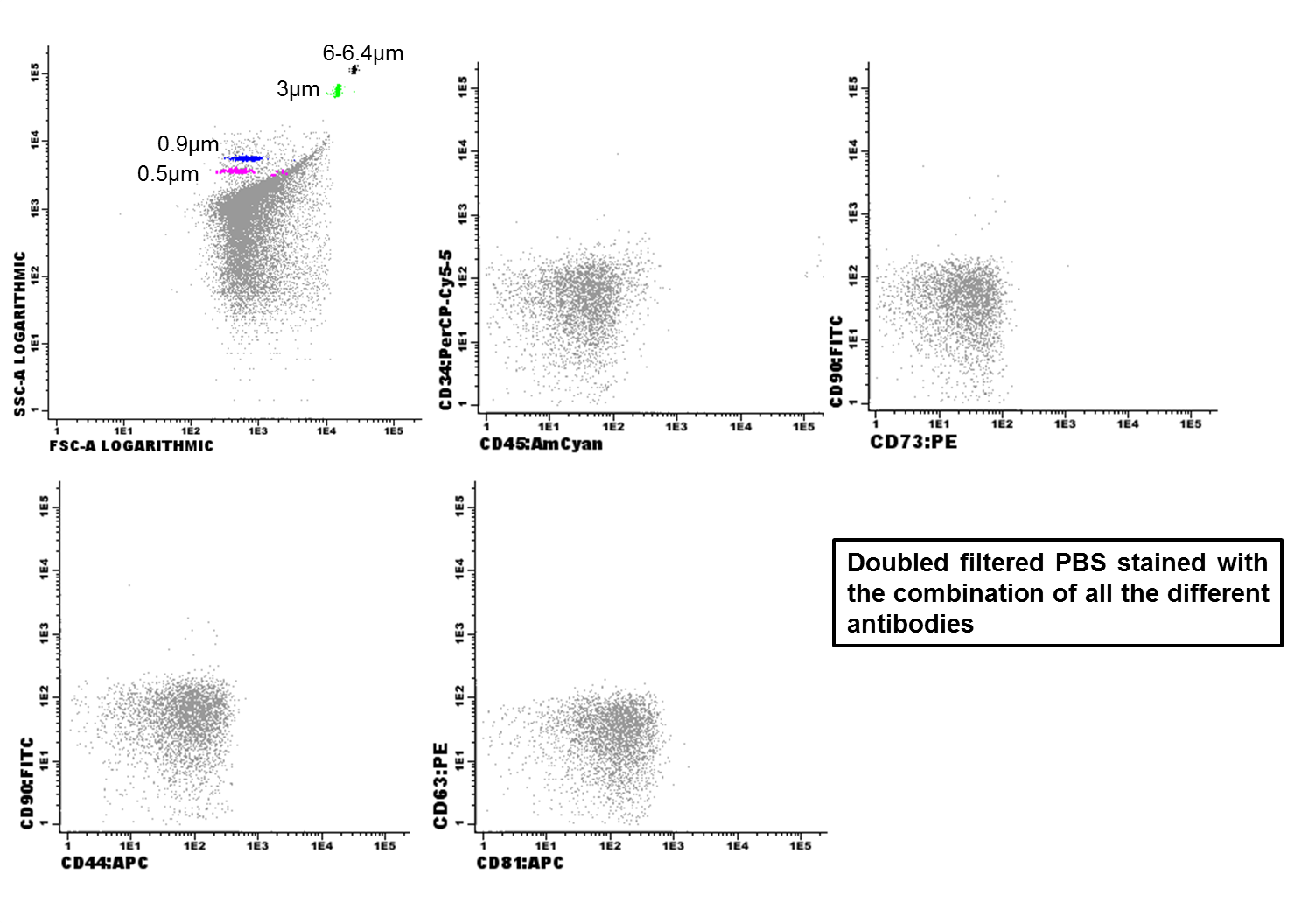

Supplement: Additional file 2: Figure S3. — Multiparametric FCM PBS dotplots. Doubled filtered PBS mixed with a combination of all different antibodies. (TIF 315 kb) [file 12964_2015_124_MOESM2_ESM.tif]

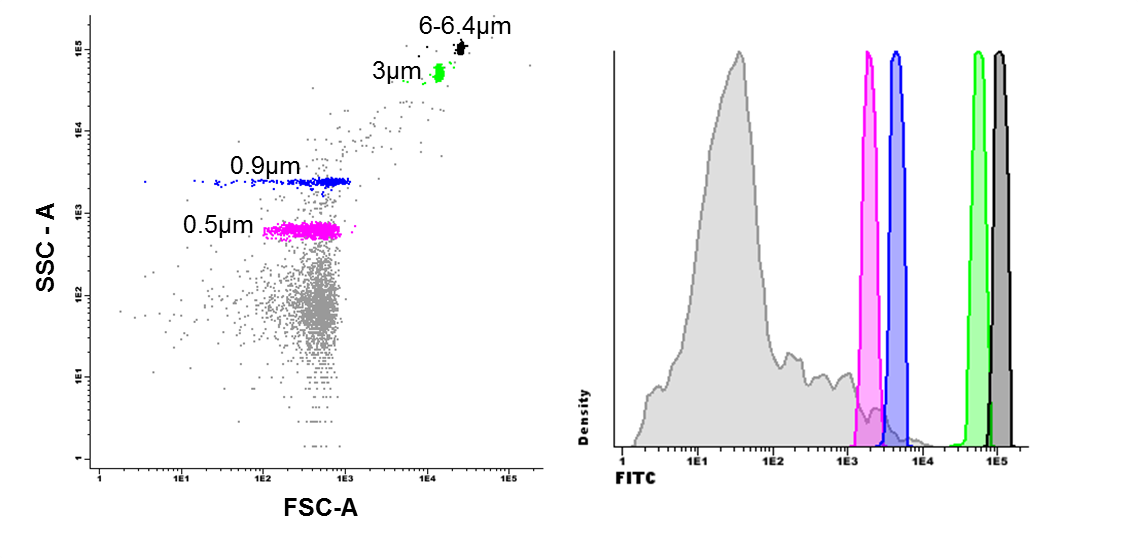

Supplement: Additional file 3: Figure S1. — Detection by FCM of different size beads. Dotplot of Megamix (0.5, 0.9 and 3 μm) and 6–6.5 μm beads dotplot (A) and density histogram (B). (TIF 120 kb) [file 12964_2015_124_MOESM3_ESM.tif]

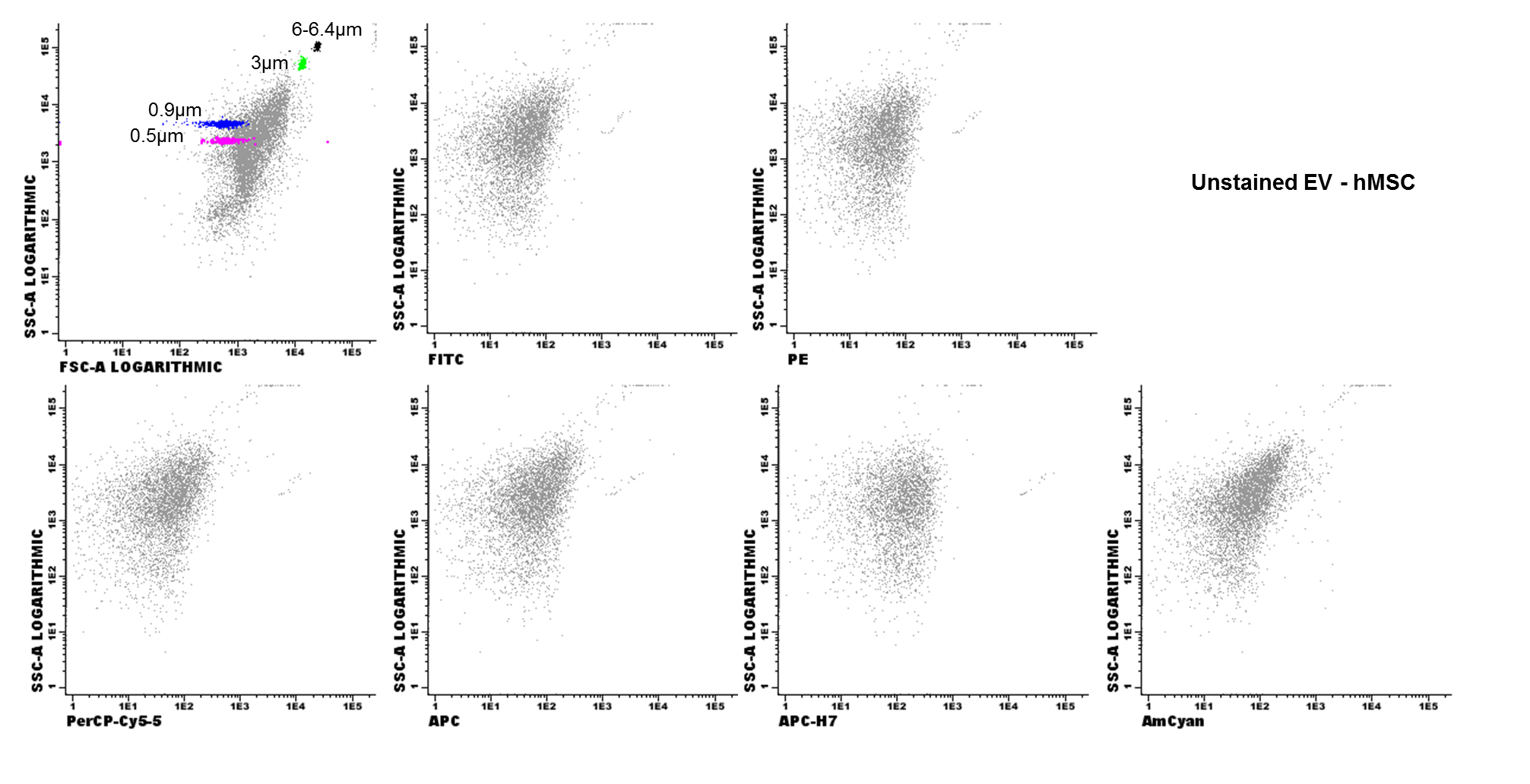

Supplement: Additional file 4: Figure S4. — Unstained hMSC-EV dotplots. (TIF 494 kb) [file 12964_2015_124_MOESM4_ESM.tif]

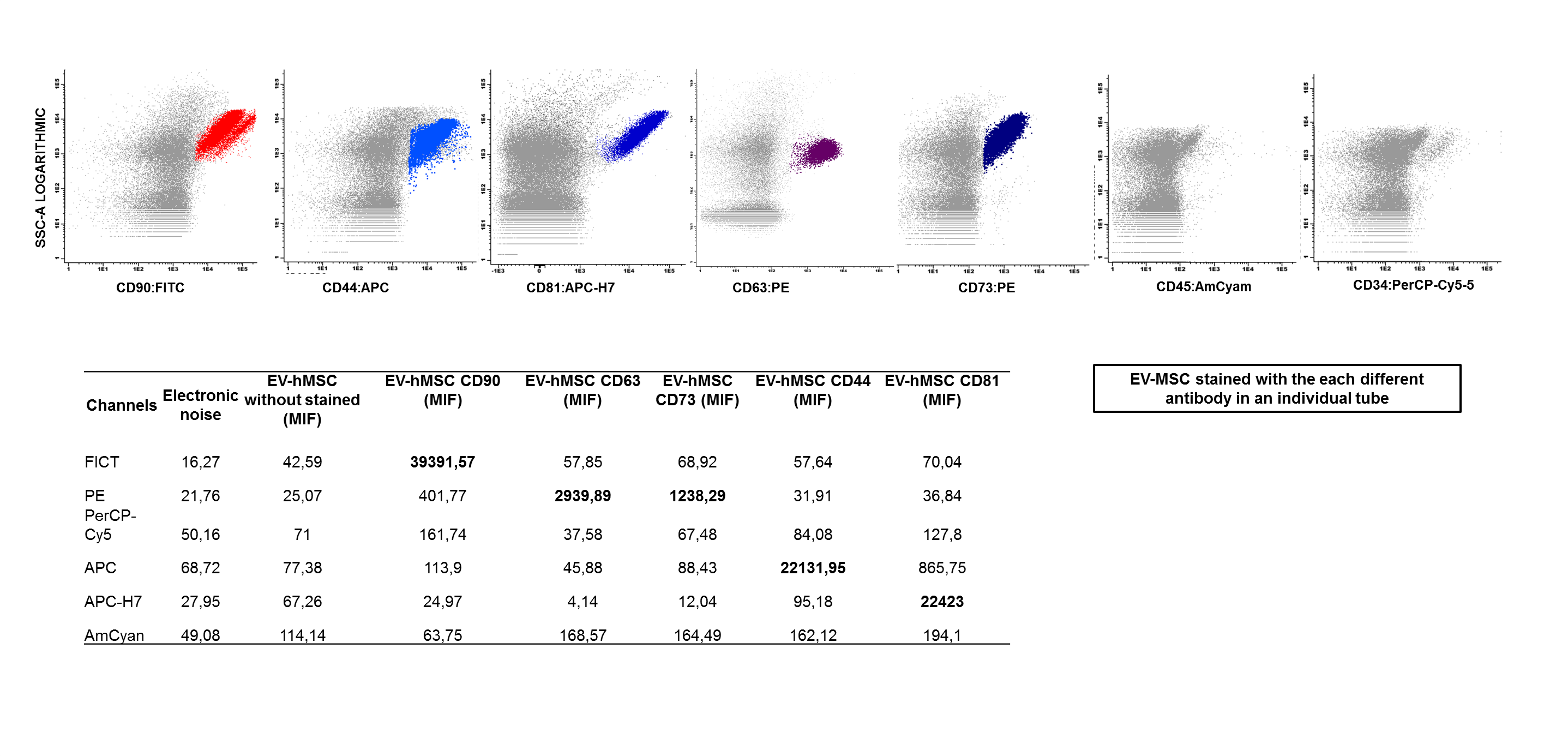

Supplement: Additional file 5: Figure S5. — Representative individual dotplots of hMSC-EV. (A) Dotplots of hMSC-EV stained with each different antibody per tube. (B) Mean fluorescence intensity (MIF) values of the different channels. (TIF 633 kb) [file 12964_2015_124_MOESM5_ESM.tif]
